# Cerebellar degeneration in gluten ataxia is linked to microglial activation

**DOI:** 10.1093/braincomms/fcae078

**Published:** 2024-03-07

**Authors:** Mara-Luciana Floare, Stephen B Wharton, Julie E Simpson, Daniel Aeschlimann, Nigel Hoggard, Marios Hadjivassiliou

**Affiliations:** Sheffield Institute for Translational Neuroscience, The University of Sheffield, Sheffield S10 2HQ, UK; Sheffield Institute for Translational Neuroscience, The University of Sheffield, Sheffield S10 2HQ, UK; Sheffield Institute for Translational Neuroscience, The University of Sheffield, Sheffield S10 2HQ, UK; Matrix Biology and Tissue Repair Research Unit, College of Biomedical and Life Sciences, School of Dentistry, Cardiff University, Cardiff CF14 4XY, UK; Department of Infection, Immunity and Cardiovascular Disease, University of Sheffield, Sheffield S10 2JF, UK; Academic Department of Neuroscience, Sheffield Teaching Hospitals NHS Trust, Royal Hallamshire Hospital, Sheffield S10 2JF, UK

**Keywords:** gluten ataxia, gluten sensitivity, neuroimmune responses, MHC-II, neuroinflammation

## Abstract

Gluten sensitivity has long been recognized exclusively for its gastrointestinal involvement; however, more recent research provides evidence for the existence of neurological manifestations that can appear in combination with or independent of the small bowel manifestations. Amongst all neurological manifestations of gluten sensitivity, gluten ataxia is the most commonly occurring one, accounting for up to 40% of cases of idiopathic sporadic ataxia. However, despite its prevalence, its neuropathological basis is still poorly defined. Here, we provide a neuropathological characterization of gluten ataxia and compare the presence of neuroinflammatory markers glial fibrillary acidic protein, ionized calcium-binding adaptor molecule 1, major histocompatibility complex II and cluster of differentiation 68 in the central nervous system of four gluten ataxia cases to five ataxia controls and seven neurologically healthy controls. Our results demonstrate that severe cerebellar atrophy, cluster of differentiation 20^+^ and cluster of differentiation 8^+^ lymphocytic infiltration in the cerebellar grey and white matter and a significant upregulation of microglial immune activation in the cerebellar granular layer, molecular layer and cerebellar white matter are features of gluten ataxia, providing evidence for the involvement of both cellular and humoral immune-mediated processes in gluten ataxia pathogenesis.

## Teaser

Floare *et al.* report severe cerebellar atrophy, microglial immune activation and lymphocytic infiltration in the cerebellum of gluten ataxia patients and conclude that both cellular and humoral immune-mediated processes are involved in the pathogenesis of gluten ataxia.

## Graphical Abstract

**Graphical Abstract:**
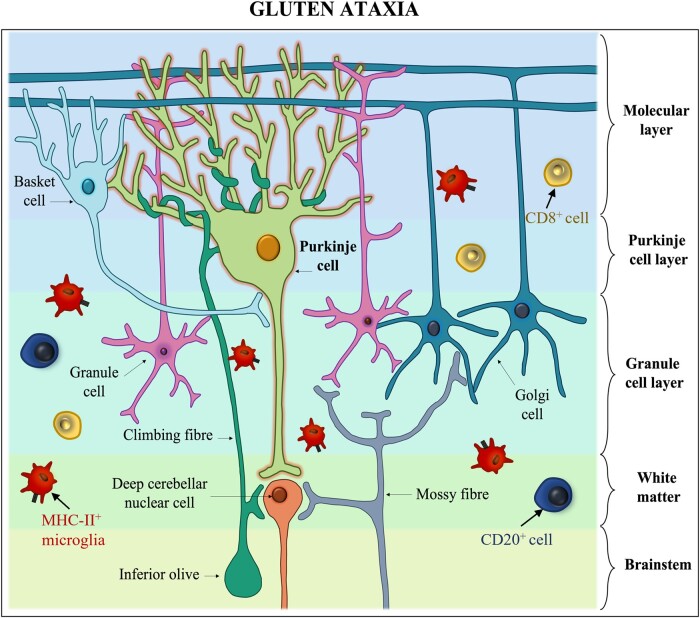

## Introduction

Gluten-related disorders are a group of immune-mediated diseases that are triggered and progress in response to gluten consumption.^1^ Whilst gluten sensitivity is most commonly associated with coeliac disease (CD), the manifestations of gluten-related disorders extend outside of the gastrointestinal tract and can affect the skin, causing dermatitis herpetiformis, and the CNS, leading to a diverse range of neurological dysfunction including gluten ataxia (GA), sensory ganglionopathy, sensorimotor axonal neuropathy, encephalopathy, myopathy, myelopathy and brain white matter abnormalities.^2-5^ Such manifestations can occur in combination with or independent of the classical small bowel lesions that define CD,^6^ making them a disease entity of their own and not a neurological manifestation of CD.

GA, the most prevalent neurological manifestation, is defined as idiopathic sporadic ataxia with positive circulating anti-gliadin antibodies in the absence of an alternative aetiology.^7^ It is clinically characterized by gait ataxia, sometimes associated with symptoms of peripheral neuropathy^6^ and more rarely with myoclonus, palatal tremor and opsoclonus myoclonus.^8,9^ There is also a rare, rapidly progressive presentation, resembling paraneoplastic cerebellar degeneration (PCD).^10^ Although neuropathological studies have been limited to date, it has been proposed that loss of Purkinje cells (PCs) throughout the cerebellar cortex is the primary neuropathological hallmark of GA.^11^ This appears in association with astrogliosis of the cerebellar white matter, vacuolation of the neuropil, perivascular cuffing with inflammatory cells and the presence of a diffuse infiltrate of T lymphocytes^7,12,13^ Extra-cerebellar pathology includes neuronal loss and gliosis in the cerebrum, inferior olives, thalamus and hypothalamus, as well as demyelination of the posterior columns and anterolateral columns of the spinal cord.^14^

GA is the commonest cause of progressive ataxia,^15^ and up to 47% of patients presenting with the classical symptoms of CD to gastroenterologists have abnormal MR spectroscopy of the cerebellum.^16^ Less than 10% of GA patients present with gastrointestinal symptoms; however, 33% show evidence of enteropathy on duodenal biopsies,^1^ whilst more than 50% of newly diagnosed CD patients show neurological dysfunction upon clinical evaluation. This raises the question of the extent of overlap between the autoimmune response responsible for CD and GA.

The ingestion of gluten and the consequential accumulation of gliadin peptides along the intestinal epithelial barrier compromises the integrity of the tight junction system,^17^ enhancing the passage of gliadin into the lamina propria, where it is deamidated by transglutaminase 2 (TG2), the autoantigen involved in CD pathogenesis. Some of the resulting deamidated gliadin peptides are highly immunogenic and are presented to CD4^+^ T cells in a major histocompatibility complex II (MHC-II)-dependent manner,^18^ triggering the release of pro-inflammatory cytokines. The activation of B cells involved in the production of antibodies against TG2/endomysium and gliadin/deamidated gliadin is driven by gluten-specific T cells.^19^ Uniquely, stable thioester-linked enzyme–gliadin peptide complexes enable gluten peptide presentation by TG-specific B cells, bringing about the failure of self-tolerance.^20^ Interestingly, in a subset of CD patients, the immune response initiated by TG2 can become systemic and circulating IgA antibodies targeting the skin resident TG3 deposit at the level of the papillary dermis.^21^ This results in an immune response leading to purpuric, vesicular lesions on the elbows, knees and buttocks that characterize dermatitis herpetiformis, the skin manifestation of gluten sensitivity.^22^ Despite extensive research, the pathogenesis of the neurological manifestations of gluten sensitivity is poorly understood.

Akin to TG2 in CD, TG6, a TG isozyme specifically expressed in the brain, has been suggested to be the primary autoantigen in GA,^6^ and the presence of circulating anti-TG6 antibodies is a potential biomarker for GA.^23,24^ Furthermore, adherence to a gluten-free diet (GFD), the main therapeutic strategy currently available for patients, is associated with reduced antibody titres^24^ and improvement of ataxia symptoms.^25^

Advancing our understanding of the extent and nature of CNS damage in GA and the mechanisms by which gluten intake progresses into the neuropathological signature of GA could facilitate diagnosis at earlier stages, when the neuronal damage could be reversible. Therefore, in the current study, we performed an extensive histopathological characterization of several CNS regions in GA, aiming to better define the neuropathological basis of gluten-related brain disease and the cellular neuroinflammatory responses associated with GA.

## Materials and methods

### Study cohort

Post-mortem (PM) human CNS tissue was obtained from the Sheffield Brain Tissue Bank, following ethical approval (REC19/SS/0029). Data were collected from a total of four patients with GA (based on the definition of GA as mentioned in the introduction), five patients with other forms of ataxia (three patients with cerebellar variant of multiple system atrophy, confirmed at PM, one patient with genetically confirmed spinocerebellar ataxia type 2 and one with genetically confirmed Friedreich’s ataxia) who represented the ataxia disease control group and eight neurologically healthy controls (Supplementary Table 1). The extensive investigations to identify the cause of the ataxia in all cases reported here can be found in our previous publication in which we described the aetiology of ataxia in 1500 consecutive cases.^15^ Immunohistochemistry for neurodegenerative markers (tau, amyloid, synuclein, TAR DNA-binding protein 43) (Supplementary Table 2) was performed as part of the diagnostic neuropathology workup using standard protocols in a diagnostic laboratory at the time of case donation.

### Haematoxylin and eosin staining and immunohistochemistry

Paraffin-embedded formalin-fixed tissue sections were stained with haematoxylin and eosin. Immunohistochemistry was performed using a standard avidin–biotin complex, and the signal was visualized using diaminobenzidine (Vector Laboratories, USA). Isotype controls were used for the antigen detection signal optimization of each primary antibody. Sections incubated in the absence of the primary antibodies were subsequently used as a negative control and were included in every run. A summary of the primary antibodies used and their conditions can be found in Supplementary Table 3. Immunohistochemistry preparations for the T-cell markers CD3, CD4 and CD8 and B-cell marker CD20 were performed by the Pathology Department at The Sheffield Teaching Hospital NHS Foundation Trust. A summary of the antibodies used can be found in Supplementary Table 2.

### Image analysis

Image analysis was performed blind to the clinical information. Selected regions of interest for the spinal cord (anterior horn, lateral corticospinal tract and the dorsal column), pons and the thalamus were marked on haematoxylin and eosin sections and mapped onto consecutive immunostained slides. Assessment of antigen-specific immunoreactivity was performed by capturing 20× bright-field microscopic images (Olympus Cell R, Olympus Biosystems, Watford, UK) in five random fields selected within the areas of interest. For the parietal cortex, images were taken across the entire cortical thickness in three adjacent transects. To quantitatively assess the immunoreactive profile of the candidate markers across the region of interest, the colour threshold was set, and the percentage area immunoreactivity exceeding the threshold was determined using analySIS D software (Olympus Biosystems, Watford, UK). The number of ionized calcium-binding adaptor molecule 1 (Iba-1)- or MHC-II-positive microglia was calculated based on size exclusion (250 pixels for MHC-II; 150 pixels for Iba-1).^26^

### Statistical analysis

Statistical analysis of the expression of neuroinflammatory markers was performed using Prism GraphPad software (GraphPad Software, Inc., USA). A non-parametric Kruskal–Wallis test followed by Dunn’s multiple comparison *post hoc* test was performed to determine the variation in neuroinflammatory markers between the study groups investigated. To determine significance, all tests were performed two-tailed and significant *P*-values were <0.05.

## Results

### Clinical history of GA cases

#### Case 1

A 62-year-old man presented with an 18-month history of tremor, ataxia and dysarthria. On examination, he had bilateral cerebellar signs with past pointing on finger nose test and heel-to-shin ataxia. He also had difficulty tandem walking. The tremor was thought to be cerebellar in origin. CT scan of the brain revealed cerebellar atrophy. Lumbar puncture demonstrated some lymphocytosis, but cytology was normal. Blood tests showed low serum B12 level and positive anti-gliadin antibodies suggestive of gluten sensitivity. Gastroscopy and gastric biopsy were normal, but unfortunately, he did not undergo duodenal biopsy. Whilst hospitalized, he suffered two bouts of aspiration pneumonia and was fitted with a percutaneous endoscopic gastrostomy tube. The patient died 1 month after being discharged at home. He never started a GFD.

#### Case 2

A 71-year-old man presented with a history of progressive ataxia. Neurophysiology confirmed sensorimotor axonal neuropathy. Blood investigations showed elevated anti-glutamic acid decarboxylase antibodies suggestive of an immune-mediated ataxia. MRI brain including MR spectroscopy of the cerebellum showed evidence of reduced metabolite level (N-acetyl-aspartate to creatine) in the vermis. His identical twin brother also developed ataxia at the same age and was found to have CD. Genetic testing using next-generation sequencing was negative for gene mutations associated with inherited ataxias. He was treated with various immunosuppressive drugs including mycophenolate, azathioprine and cyclophosphamide, with variable benefit. Further serological testing using a newly introduced anti-gliadin assay (6 years later) showed him to be positive for anti-gliadin antibodies (previous tests using other assays were normal). The duodenal biopsy was normal. He refused to go on a GFD. He continued to progress with worsening ataxia and ended up in a nursing home bedbound. He died at the age of 80. The last brain imaging done 7 years after the original presentation showed a significant decline in the MR spectroscopy of the cerebellum in keeping with his clinical deterioration.

#### Case 3

A 49-year-old woman presented with proximal weakness. Neurophysiological assessment and subsequent muscle biopsy were suggestive of polymyositis, and she was treated with steroids. She remained well until the age of 62 when she was diagnosed with CD on duodenal biopsy, following episodes of diarrhoea. Two years later, she presented with distal sensory symptoms and unsteadiness of gait. Neurophysiology was suggestive of a sensory neuropathy with some myopathic features. Repeat muscle biopsy showed a myopathic picture but no active inflammation. The dose of steroids was nonetheless increased but without any obvious clinical improvement. Her anti-gliadin antibodies were positive despite a GFD. A repeat duodenal biopsy showed crypt hyperplasia and increased intra-epithelial lymphocytes, suggestive of ongoing exposure to gluten. There was no suggestion of refractory CD. She remained unwell with some weight loss and progressive ataxia. Her biochemical profile suggested malabsorption (low calcium, magnesium, vitamin D and low albumin). The patient died at home aged 67 a few days after the last clinic review.

#### Case 4

**Figure 1:**
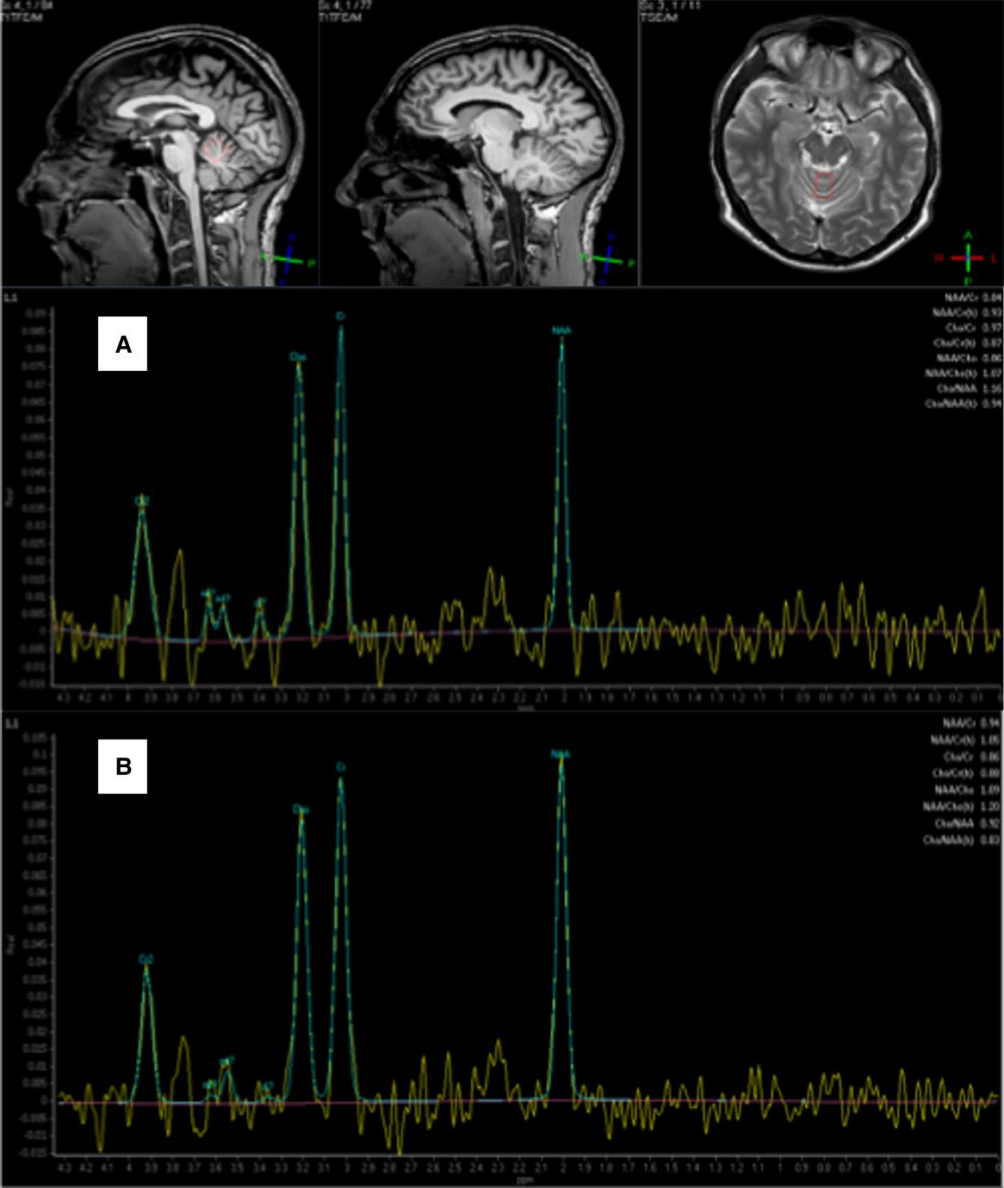
**MR spectroscopy of the vermis of the cerebellum from Patient 4.** (**A**) Spectroscopy A was obtained at presentation of ataxia and showed reduced N-acetyl-aspartate to creatine area ratio at 0.84 (normal range is >1). There was no evidence of cerebellar atrophy. (**B**) Spectroscopy B was obtained several years later whilst the patient was on a strict GFD. The N-acetyl-aspartate to creatine ratio shows an increase to 0.94, an observation that is commonly seen in patients who embark on strict GFD.

A 39-year-old man presented with an 18-month history of clumsiness and gait instability. He was no longer able to cycle and felt that his speech was at times slurred. Examination showed evidence of cerebellar ataxia with impaired finger nose and heel-to-shin testing. His gait was abnormal as he was rather unsteady to the point of having to use a wheelchair. In addition, he had evidence of an irregular tremor of his arms and head suggestive of myoclonus. Initial investigations showed IgA deficiency. He was positive for IgG anti-gliadin antibodies. Duodenal biopsy showed no evidence of CD. He was diagnosed with GA. MRI revealed no cerebellar atrophy. However, MR spectroscopy of the cerebellum was abnormal. He was treated with clonazepam and a GFD. Nerve conduction investigations showed no evidence of neuropathy or sensory ganglionopathy, but there was ongoing severe pain over his legs and arms. The possibility of small fibre neuropathy was raised, and he was found to have abnormal thermal thresholds in keeping with small fibre neuropathy. His ataxia stabilized on GFD and MR spectroscopy of the cerebellum improved (Fig. 1). The pain related to the small fibre neuropathy remained very prominent and disabling. He was found dead in his bed at the age of 51 years old.

### Histopathology of the CNS in GA

All four of the cases in which a diagnosis of GA was made in life underwent PM neuropathological assessment, with retention of the brain and examination following formalin fixation.

#### Cerebellum

**Figure 2:**
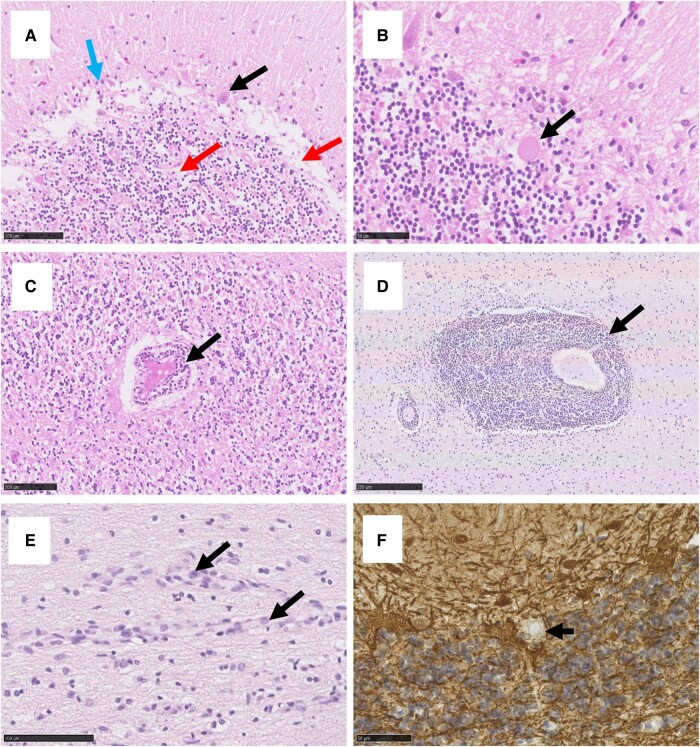
**Histological examination of cerebellum in GA.** The cerebellum showed atrophy with subtotal loss of PCs (black arrow in **A**) and Bergmann gliosis (blue arrow in **A**). This was accompanied by attenuation of the GL (red arrow in **A**) and the presence of eosinophilic structures (**B**) and lymphocytic cuffs around blood vessels (**C**) in Case 1. The white matter of Case 1 displayed perivascular cuffing (**D**) and hyperplasia of endothelial cells (**E**). Immunohistochemistry to neurofilament protein displayed empty baskets where PCs were lost but surrounding axonal terminals remained (**F**). Scale bar represents 50 (**B**, **F**), 100 (**A**, **C**, **E**) and 250 µm (**D**).

**Figure 3:**
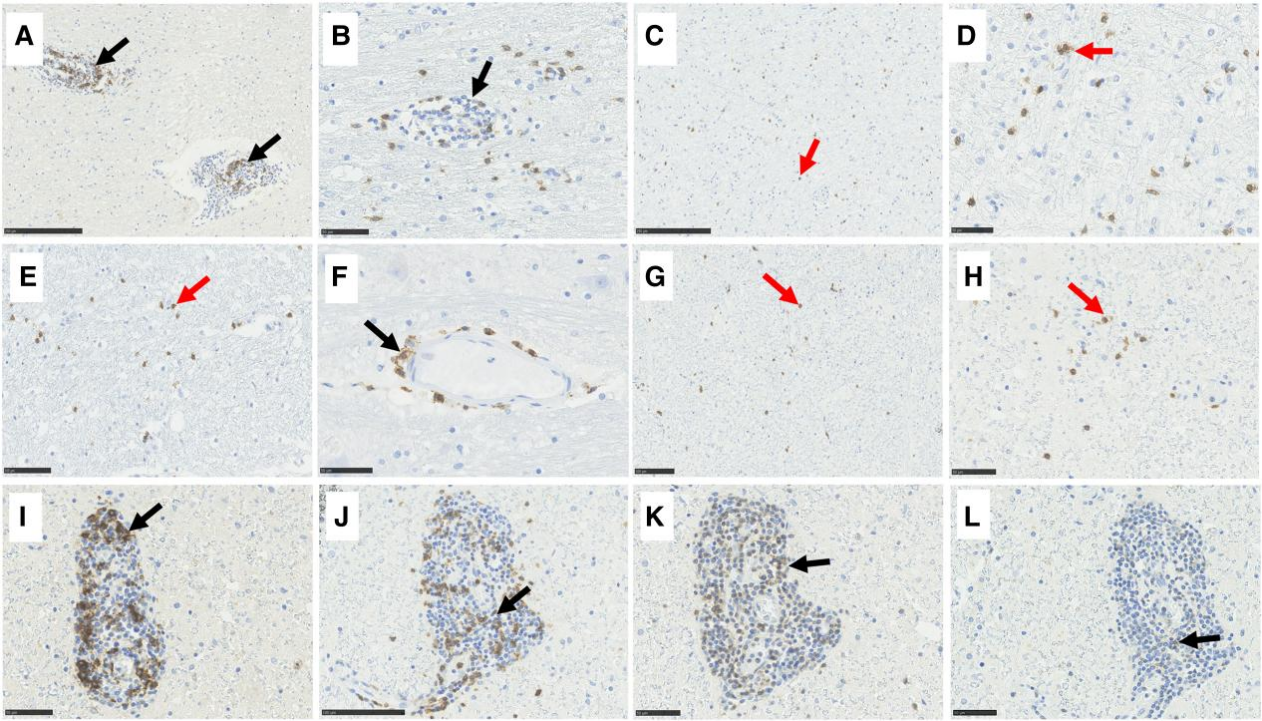
**Variation in the immunoreactive profile of B-cell and T-cell markers in GA.** The cerebellar white matter displayed perivascular cuffing with numerous CD20^+^ cells (**A**) and moderate CD8^+^ cells (**B**) and a dense infiltrate of CD8^+^ cells in the parenchyma (red arrow in **C**, **D**). The superior cerebellar peduncles of Case 1 showed moderate infiltrate of CD8^+^ cells (**E**) whilst Case 2 displayed occasional cells positive to CD8 perivascularly in the basis pontis (**F**). Occasional cells positive to CD8 (**G**, **H**) were observed in the dorsal column, together with a strong perivascular lymphocytic infiltrate of CD20 (**I**), CD8 (**J**) and CD3 (**K**) cells. CD4^+^ cells were only rarely seen in the spinal cord of Case 1 (**L**). Scale bar represents 50 (**B**, **D**, **F**, **H**, **I**, **K**), 100 (**E**, **G**, **J**) and 250 μm (**A**, **C**).

Histological examination of the cerebellum showed atrophy with subtotal loss of PCs and Bergmann gliosis (Fig. 2A), both at the deep and superficial surface of the cerebellar cortex in Cases 1 and 2 (Supplementary Table 4). This was accompanied by attenuation of the molecular (ML) and granular layer (GL) (Fig. 2A). Case 1 showed the presence of pale eosinophilic structures (Fig. 2B) and lymphocytic cuffs around vessels within the GL (Fig. 2C). Perivascular space widening was present in the GL in Case 2. Case 3 showed very mild cerebellar atrophy with mild patchy loss of PCs and Bergmann glia, whilst no atrophy was present in Case 4. The white matter displayed areas of pallor, which in Cases 1 and 4 was accompanied by perivascular cuffing (Fig. 2D) with numerous CD20^+^ cells (Fig. 3A) and moderate numbers of CD8^+^ cells (Fig. 3B). Additionally, a dense infiltrate of CD8^+^ cells was present in the white matter in Cases 1 and 3 (Fig. 3C). For Case 1, white matter pallor was most prominent adjacent to the dentate nucleus. Endothelial cells appeared plumped and reactive, but no increase in cell numbers or vascular necrosis was observed (Fig. 2E). Immunohistochemistry to neurofilament protein in Case 2 displayed many empty baskets where PCs were lost but surrounding axonal terminals remained (Fig. 2F). This was accompanied by an isolated axonal swelling. Additionally, there was mild lymphocytic cuffing around a medium vessel in the dentate nucleus of Case 2.

#### Brainstem

**Figure 4:**
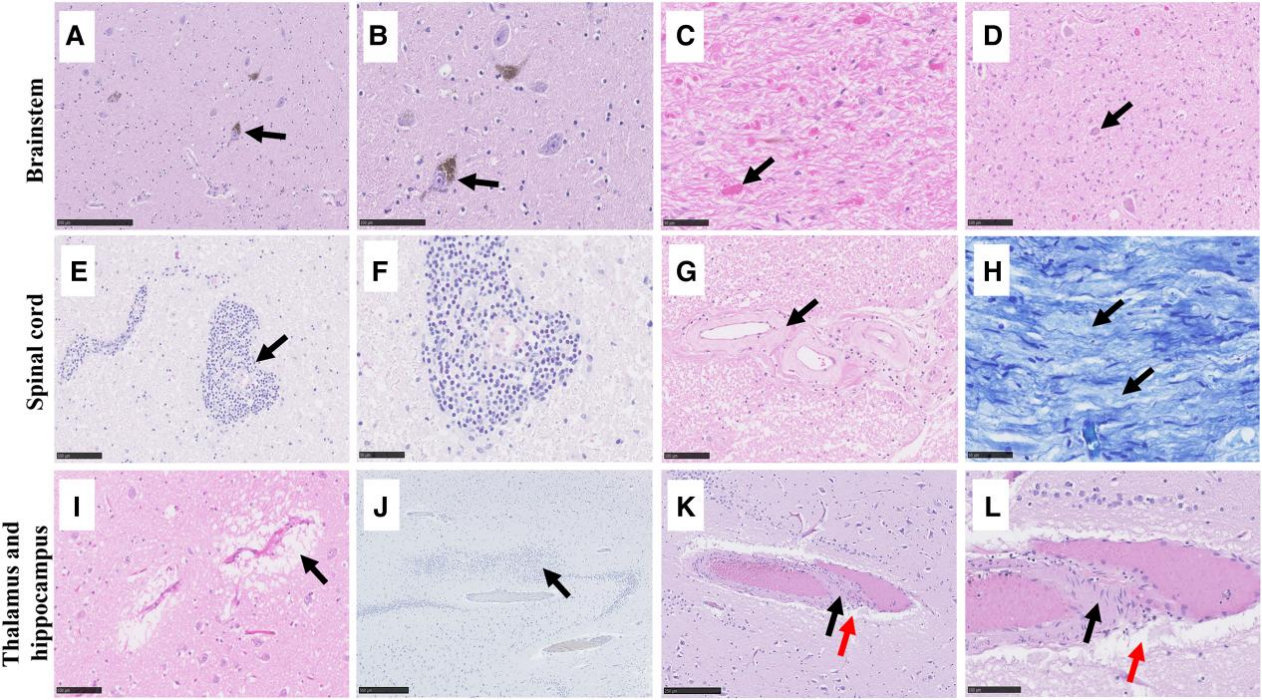
**Histological examination of the extra-cerebellar CNS in GA.** Mild cell loss was observed in the substantia nigra of Case 2 (**A**, **B**).The gracile and cuneate nuclei in the medulla of Case 3 displayed Rosenthal fibres (**C**) and neuronal loss (**D**). Dense perivascular lymphocytic infiltration (**E**, **F**) and sclerotic vessels (**G**) were observed in the dorsal column of the spinal cord, whilst mild patchy loss of myelin was observed in peripheral nerve roots (**H**). Perivascular space widening was a common feature of thalamic vessels across all cases (**I**). The dentate layer appeared reduplicated in the hippocampus of Case 2 (**J**) and sclerotic vessels were observed in the hilus of Case 4 (black arrow in **K** and **L**) together with perivascular space widening (red arrow in **K** and **L**). Scale bar represents 50 (**C**, **F**, **H**), 100 (**B**, **D**, **E**, **G**, **I**, **L**), 250 (**A**, **K**) and 500 μm (**J**).

Case 2 showed mild cell loss from the substantia nigra (Fig. 4A) and from the locus coeruleus. Case 1 showed a moderate infiltrate of CD8^+^ cells in the superior cerebellar peduncles (Fig. 3E), whilst Case 2 displayed occasional perivascular cells positive for CD8 in the tegmentum and the grey and white matter of the basis pontis (Fig. 3F). Sparse CD4^+^ cells were observed in the pons of Case 1. No PM material from the midbrain region was available from Case 1. No other obvious abnormalities were present in the midbrain and the pons.

In Case 3, Rosenthal fibres were scattered throughout the region of the gracile and cuneate nuclei (Fig. 4C) and pallor was present adjacent to it. There was obvious loss of neurons in the gracile and cuneate nuclei (Fig. 4D). Case 2 showed perivascular space widening and one sclerotic vessel with sparse lymphocytic infiltration. No other obvious abnormalities were present in the medulla for Cases 1, 2 and 4.

#### Spinal cord

Perivascular space widening and a strong perivascular lymphocytic infiltrate (Fig. 4E) of CD20^+^ (Fig. 3I) and CD8^+^ cells (Fig. 3J) were observed in Case 1, whilst a moderate infiltrate of CD3^+^ cells was present in the dorsal column (Fig. 3K) of the same case. Additionally, occasional cells positive for CD8 (Fig. 3G) and CD3 were observed in the parenchyma of the dorsal column, the corticospinal tract and the anterior horn of Case 1. Sparse CD4^+^ cells were observed in the spinal cord of Case 1. Sclerotic vessels were observed in Case 3 (Fig. 4G). The spinal nerve roots, cauda equina and the peripheral nerve roots showed mild patchy loss of myelin staining in both Cases 3 and 4 (Fig. 4H), and there was a sparse infiltrate of lymphocytes around some of the endoneurial and perineurial vessels in Case 4. Case 1 showed lymphocytic infiltration in the lumbar dorsal root ganglia, which was accompanied by occasional loss of dorsal root ganglion cells. For Case 2, no spinal cord PM material was available.

#### Cerebral cortex

There was pallor of the subpial surface and vacuolation in Layer 2 of the parietal cortex. However, no obvious loss of neurons was observed. For Case 1, no PM material from the parietal cortex was available.

Case 1 showed occasional perivascular lymphocytic cuffs in the frontal lobe and some perivascular haemorrhage within the white matter of the parieto-occipital lobe, particularly in the periventricular region. Additionally, there were foci of loss of ependymal lining cells and proliferation of the subependymal astrocytes. The occipital lobe of Case 2 displayed a perivascular area of white matter with pallor. No other abnormalities were observed in the rest of the cortical areas.

#### Thalamus

The thalamus displayed pallor and moderate vacuolation in Cases 2 and 4, with a sparse lymphocytic infiltrate and perivascular space widening in all cases (Fig. 4I). Additionally, in Case 1, some vessels appeared sclerotic and with mild lymphocytic cuffs.

#### Hippocampus

The hippocampus showed some reduplication of the dentate layer in Case 2 (Fig. 4J), whilst Case 1 showed occasional loss of neurons within the end folium of the hippocampus. The neuronal loss in Case 1 was accompanied by some mononuclear cell infiltration and sparse perivascular lymphocytic cuffing within the leptomeninges. There was mild vacuolation in the parahippocampal cortex in Case 4. Additionally, Case 4 displayed one sclerotic vessel in the hilus and perivascular space widening (Fig. 4K). No PM material from Case 3 was available for this region.

### Neuroinflammation is a feature of all brain regions in GA

**Figure 5:**
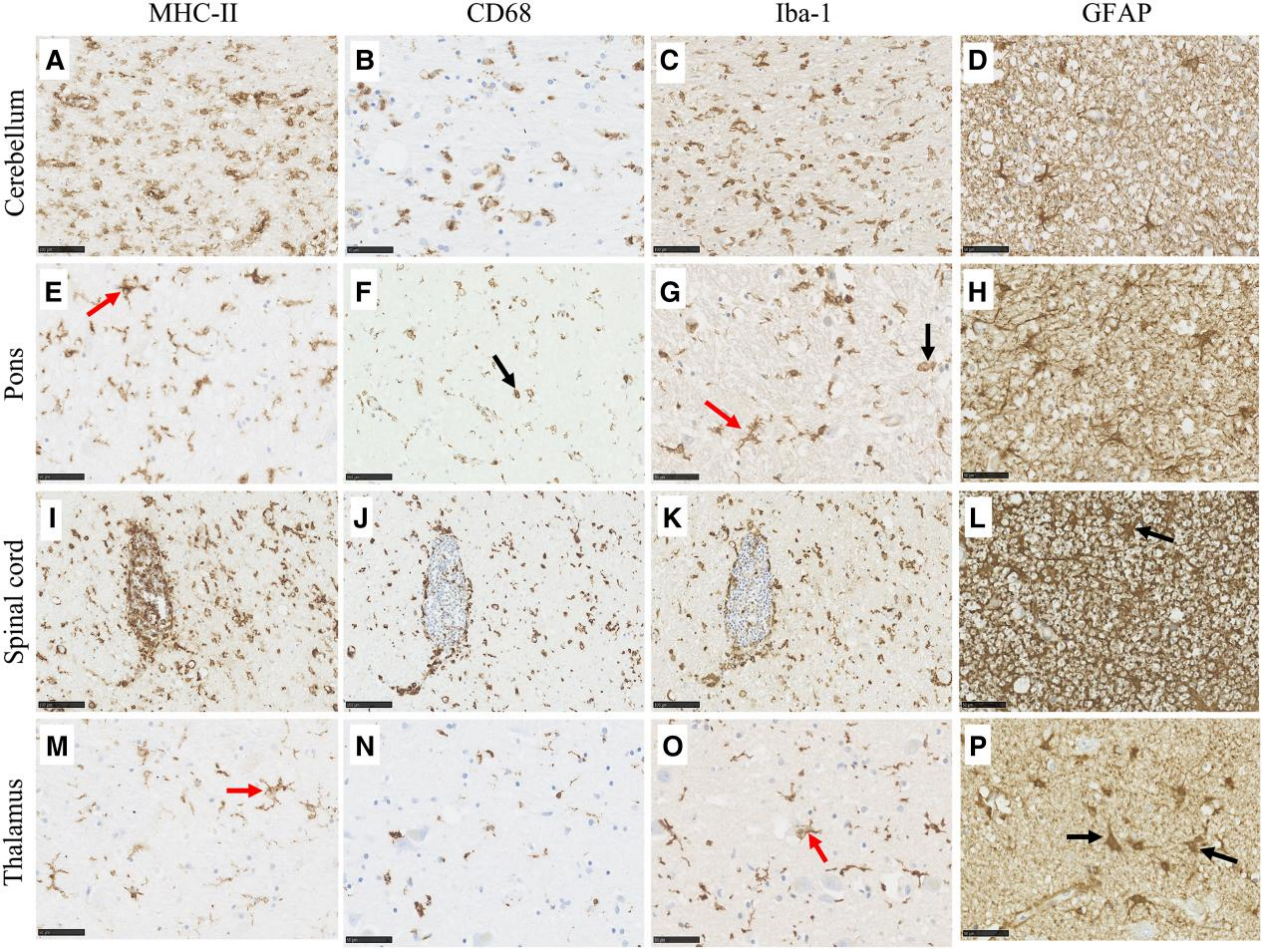
**Variation in the immunoreactive profile of glial markers in the cerebellum, pons, spinal cord and thalamus of GA patients.** Ameboid microglia positive to MHC-II (**A**), CD68, (**B**) and Iba-1 (**C**) were present in the cerebellar white matter of GA cases, together with astrogliosis (**D**). Pons immunoreactivity (second row) was most marked in the superior cerebellar peduncles where an upregulation in immunoreactive ameboid (black arrows in **F** and **G**) and hypertrophic (red arrows in **E** and **G**) microglia and glial fibrillary acidic protein + astrocytes (**H**) were observed. The spinal cord dorsal column (third row) displayed extensive microgliosis, particularly evident around dorsal column blood vessels (**I–J**) and dense astrogliosis (**L**). Astrogliosis was also present throughout the thalamus (**P**), together with hypertrophic microglia positive to MHC-II (**M**) and Iba-1 (**O**). Low levels of CD68 immunoreactivity were observed in the thalamus (**N**). Scale bar represents 50 (**B**, **D**, **E**, **G**, **H**, **L**, **M–P**) and 100 μm (**A**, **C**, **F**, **I–K**).

The immunoreactive profile of glial fibrillary acidic protein demonstrated Bergmann gliosis in the PC layer and extensive astrocytic gliosis in the GL and cerebellar white matter in Cases 1, 2 and 3 (Fig. 5D). For Case 4, this favoured the grey/white matter interface. MHC-II, cluster of differentiation 68 (CD68) and Iba-1 detection demonstrated extensive microglial proliferation and activation with numerous ameboid microglia in the cerebellar white matter of Cases 1 and 2 (Fig. 5A–C). Throughout the cerebellar grey and white matter, microglia were observed in various morphologies ranging from hypertrophic to ameboid.

Dense astrogliosis was also present in the pons (Fig. 5H), the grey and white matter of the spinal cord (Fig. 5L), the subpial region of the parietal cortex, the cortical white matter and the thalamus (Fig. 5P). Additionally, the CA1–CA4 hippocampal regions in Case 4, as well as the subpial layers of the entorhinal cortex and occipitotemporal gyrus, the white matter of the parahippocampal gyrus and the fornix displayed mild to moderate astrogliosis.

This was accompanied by microgliosis with ameboid and hypertrophic microglia in the superior cerebellar peduncles of the pons (Fig. 5E–G), the dorsal column and cervical cord grey matter of the spinal cord (Fig. 5I–K) and the thalamus (Fig. 5M–O). For Case 1, microgliosis of the dorsal column was particularly marked around blood vessels (Fig. 5I–K). Additionally, Case 3 showed an unusual pattern of reactivity for Iba-1 in which Iba-1 upregulation was present around the spinal cord and towards the surface. Mild to no microgliosis was observed in the other regions of the pons and the spinal cord, the parietal cortex and the thalamus.

To further substantiate that neuroinflammation is a significant feature of ataxia and identify whether this is associated with the expansion of a specific subset of cells, expression of these glial markers was quantified by image analysis and compared with a neurologically healthy control group and an ataxia control group with non-gluten forms of ataxia (Supplementary Table 1).

### The cerebellum of GA cases contains significantly higher levels of MHC-II-expressing activated microglia

**Figure 6:**
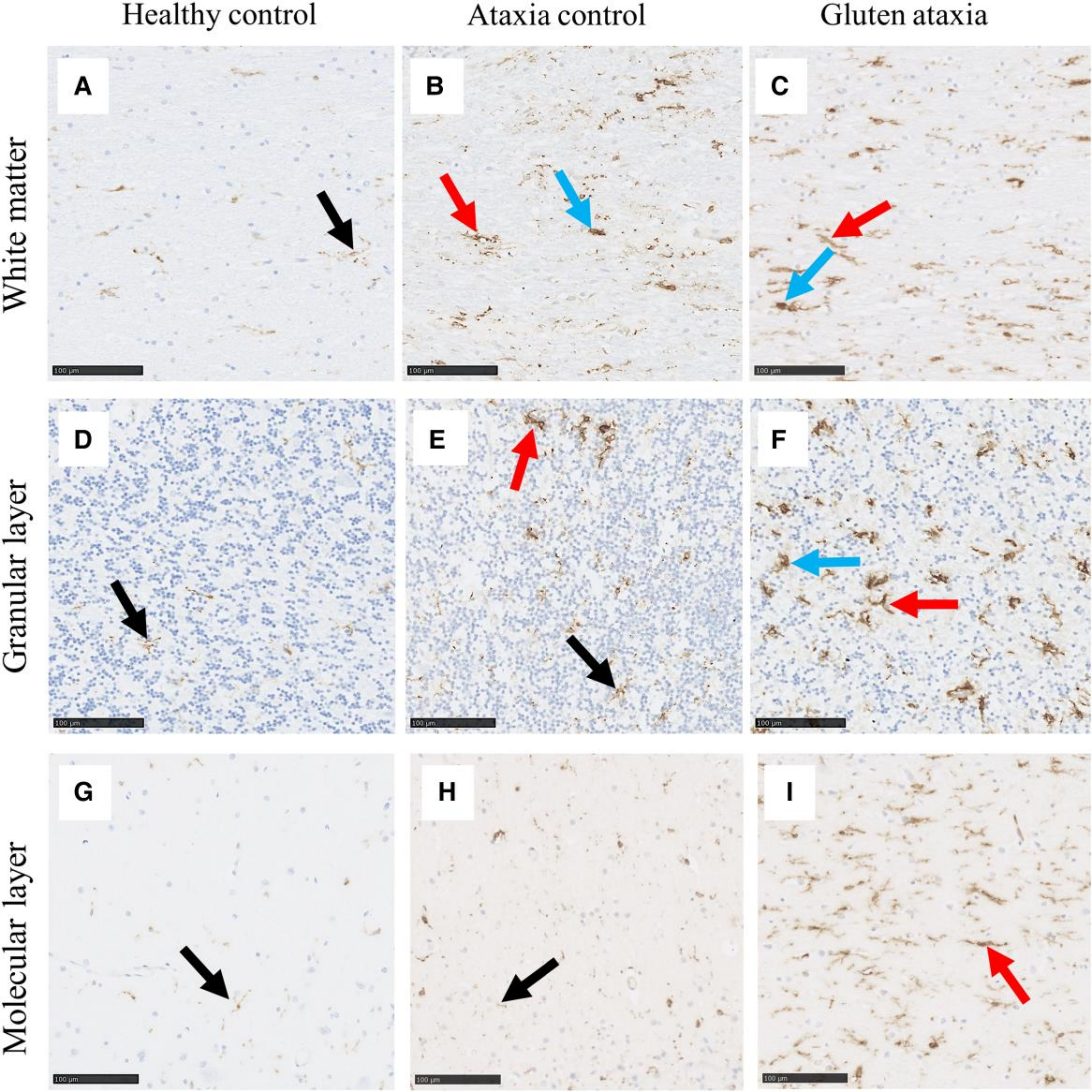
**Comparison in the immunoreactive profile of MHC-II in the cerebellum between groups.** In the neurologically healthy control group, MHC-II^+^ microglia were observed in a ramified phenotype in both the cerebellar white matter (**A**), as well as the GL (**D**) and the ML (**G**) of the cerebellar cortex. Hypertrophic (red arrow in **B** and **C**) and ameboid (blue arrow in **B** and **C**) MHC-II^+^ microglia were frequently observed in the white matter of AC (**B**, **E**) and GA (**C**, **F**) cases. In the GL (**E**) and ML (**H**) of the AC group, MHC-II^+^ microglia were mostly seen in a ramified phenotype (black arrow in **E** and **H**), with sparse hypertrophic microglia (red arrow in **E**) distributed across the cerebellar cortex. In contrast, hypertrophic MHC-II^+^ microglia were most abundant in both the GL (red arrow in **F**) and ML (**I**) of GA cases. Additionally, sparse ameboid MHC-II^+^ microglia were observed in the GL of GA cases (blue arrow in **F**). Scale bar represents 100 µm (**A–I**).

**Figure 7:**
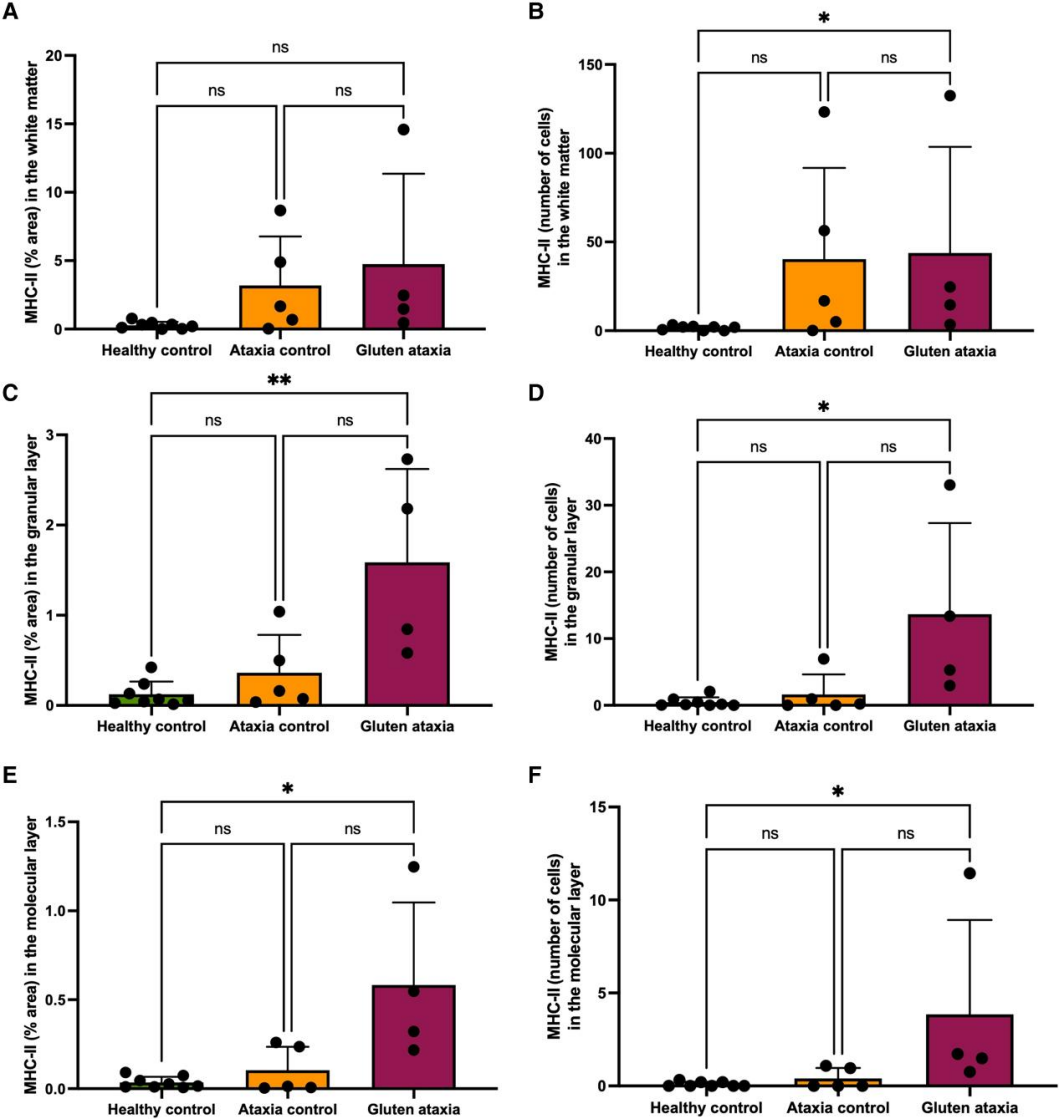
**Immunoreactive profile of MHC-II in the cerebellar cortex and white matter.** A significant increase in the percentage area immunoreactivity to MHC-II was observed in the GL (*P* = 0.0095 for Kruskal–Wallis test) (**C**) and ML (*P* = 0.0325 for Kruskal–Wallis test) (**E**) but not the white matter (*P* = 0.0637 for Kruskal–Wallis test) (**A**) of GA cases relative to neurologically healthy controls. Additionally, a significant increase in the number of cells positive to MHC-II was detected in the white matter (*P* = 0.0288 for Kruskal–Wallis test) (**B**), GL (*P* = 0.0218 for Kruskal–Wallis test) (**D**) and ML (*P* = 0.0118 for Kruskal–Wallis test) (**F**) of GA cases. ns, non-significant.

GA cases displayed a significant increase in MHC-II percentage area immunoreactivity in the GL (*P* = 0.0095) and the ML (*P* = 0.0325) compared with neurologically healthy controls. Likewise, significantly higher numbers of MHC-II^+^ cells were demonstrated in the GL (*P* = 0.0218), ML (*P* = 0.0118) and the white matter (*P* = 0.0288) of GA cases. No statistically significant differences were detected between the ataxia control group and HC (Figs. 6 and 7).

Case-to-case variability was high for CD68 and Iba-1 immunoreactivity amongst GA cases, and therefore, it was not possible to establish whether true differences between groups exist (Supplementary Fig. 1).

### GA patients and patients with non-immune mediated forms of ataxia display different patterns of microglial activation

**Figure 8:**
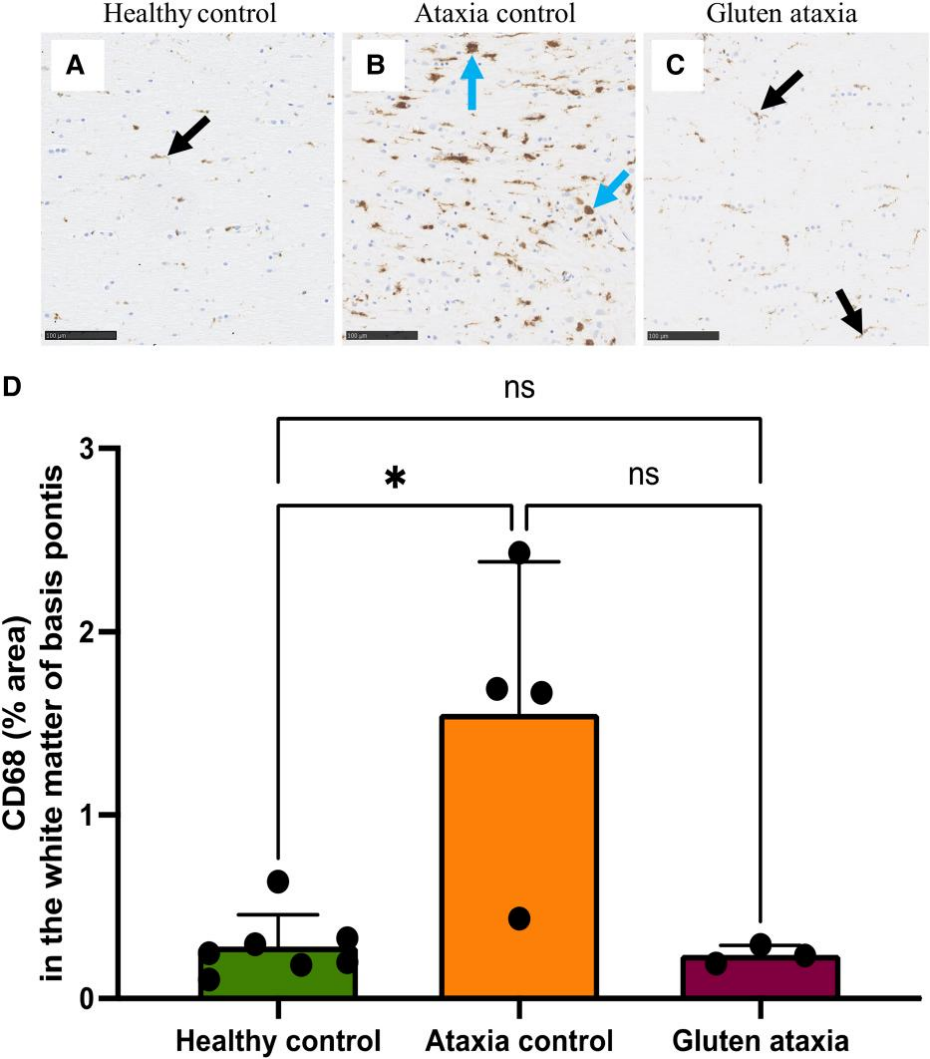
**Immunoreactive profile of CD68 in the pontine white matter.** Compared with the AC group, where a high load of CD68^+^ ameboid microglia were observed in the white matter of basis pontis (**B**), the microglia positive to CD68 in the HC (**A**) and GA (**C**) groups mainly displayed a ramified phenotype. A significant increase in CD68 immunoreactivity was measured in the basis pontis white matter of the ataxia control group compared with neurologically healthy controls (*P* = 0.0443 for Kruskal–Wallis test) but not to GA cases (*P* = 0.0698 for Kruskal–Wallis test). Scale bar represents 100 µm (**A–C**). ns, non-significant.

No changes in MHC-II, CD68 or Iba-1 expression were observed in the pons, parietal cortex, spinal cord or thalamus of GA cases compared with the healthy control group (Supplementary Table 5). This was different from the ataxia control group, which showed a significant increase in CD68 expression in the basis pontis white matter compared with neurologically healthy controls (*P* = 0.0443) (Fig. 8) and a significantly decreased expression of Iba-1 in the parietal cortex compared with neurologically healthy individuals (*P* = 0.0371 for % area; *P* = 0.0393 for number of cells) (Supplementary Fig. 2).

### Activation of astrocytes is observed in GA

A non-significant increase in glial fibrillary acidic protein expression was detected in the parietal cortex of GA patients relative to ataxia controls (*P* = 0.0505), but not compared with healthy controls (*P* = 0.4057) (Supplementary Fig. 3). No significant differences were detected in the cerebellum, spinal cord, pons and thalamus (Supplementary Table 5).

## Discussion

GA is the commonest immune-mediated form of ataxia and the primary neurological manifestation of gluten sensitivity; however, its neuropathological basis is still poorly defined. In this study, we perform an extensive histological characterization of the neuropathology of GA and investigate the possible contribution of cellular neuroinflammation resulting in neurodegeneration across five regions of the CNS. We have a well-selected collection of cases with a long clinical follow-up in which we demonstrate that GA is characterized by severe atrophy of the cerebellar cortex, lymphocytic infiltration in the cerebellar grey and white matter and a significant upregulation of microglial immune activation in the cerebellar GL and ML and white matter.

Previous clinical and histological studies of GA patients demonstrated that the cerebellum is the primary site of injury in GA^7,27^ and that evidence of neuronal loss and demyelination can be traced up to the level of the cerebrum^12^ and down to the level of the spinal cord.^28,29^ Our results support the current literature but extend our knowledge of the nature of the lymphocytic infiltrate as being primarily CD20^+^ and CD8^+^. Although histological studies of both GA and other immune-mediated cerebellar ataxias are rare, the neuropathological features of GA appear to be similar to those of primary autoimmune cerebellar ataxia, anti-glutamic acid decarboxylase ataxia and even paraneoplastic ataxia in which lymphocytic infiltration in the cerebellum and the spinal cord and degeneration of the spinocerebellar tracts and the dorsal column are prominent features.^30-32^ However, variation between individuals has been reported in most forms of immune-mediated cerebellar ataxias, including GA, and some patients can display little to no cerebellar pathology but yet be clinically ataxic.^33^ It is possible that such cases have a sensory ganglionopathy (another common neurological manifestation of gluten sensitivity) to account for the ataxia. Cerebellar atrophy with prominent loss of PCs and astrogliosis have been described in cases of myoclonic ataxia associated with CD^34^ and in cases of gluten encephalopathy,^35,36^ whilst perivascular cuffing of lymphocytes and lymphocytic infiltration can be seen in the spinal roots of peripheral nerves of patients with gluten neuropathy^37^ and gluten-related sensory ganglionopathy.^13^ In the current study, the pattern of microglial responses and the presence of CD20^+^ and CD8^+^ cells identify GA as an immune-mediated ataxia, different in its pathogenesis to other genetic or non-immune sporadic forms of ataxia.

Genetic susceptibility in the form of the HLA haplotype is thought to play a significant role in the pathogenesis of gluten-related disorders^23^ due to the ability of the MHC-II to restrictively present TG2/TG6-modified gluten-derived peptides to CD4^+^ T cells. In CD, this leads to the production of plasmacytosis, anti-TG2 antibodies and activation of intra-epithelial cytotoxic T cells in a specific context of cytokines [including interleukin 15 (IL-15) signalling], which ultimately results in gut tissue changes known as the triad of villous atrophy, crypt hyperplasia and increased intra-epithelial lymphocytes characteristic of CD.^19^ The interplay between gluten, genetics and IL-15-driven tissue inflammation in the establishment of CD pathology was highlighted in a disease mouse model.^38^ Importantly, these studies revealed that IL-15 overexpression in the gut links the systemic autoimmune response to local activation of intra-epithelial cytotoxic T cells. Hence, the absence of intestinal tissue destruction (‘normal’ gut mucosa) can be a consequence of reduced cytokine signalling even in the presence of adaptive gluten immunity and thereby explain the spectrum of presentation seen in patients. We hypothesize that a similar pathogenic mechanism is at play in patients with GA and centred in the gut. This is in line with recent findings showing that a TG3-specific B-cell response is generated in the gut in dermatitis herpetiformis patients.^39^ The mechanism leading to the degeneration of the cerebellum in GA remains to be elucidated, but our results highlight the involvement of immune-mediated processes. Innate immune surveillance in the CNS is principally coordinated by microglia.^40^ Upon activation, microglia switch from a dynamic ‘resting state’^41^ into an ‘ameboid’ phenotype, migrating towards the site of damage and exerting either neurotoxic or neuroprotective functions.^42,43^ Cerebellar microglial activation, perivascular lymphocytic cuffs and infiltration of lymphocytes within the PC layer are some of the main inflammatory changes observed in the early stages of PCD, a form of immune-mediated ataxia triggered by cancer. An increase in MHC-II expressing cells in the cerebellum was reported in a case study of PCD,^44^ supporting its immune-mediated aetiology. Additionally, neuropathological case studies have demonstrated that the infiltrating lymphocytes in the cerebellum of PCD patients are mainly CD3^+^ and CD8^+^ T cells^45,46^ and that PCD is mediated by a CD8^+^ T-cell immune response rooted in the existence of cross-reactivity between PCs and onconeural antibodies.^47-50^ Interestingly, cerebellar damage is apparently not driven by autoantibody binding (as the disease cannot be induced through adoptive transfer) but an antigen-specific T-cell response. Similarly, recent histological characterization of the cerebellum in patients with CD and idiopathic ataxia^27^ demonstrated significantly higher CD3^+^ and CD8^+^ lymphocytes count, as well as changes in microglia as assessed by Iba-1 detection in the cerebellum of GA cases compared with controls. Our results are in line with those findings and further show the presence of CD20^+^ perivascular inflammatory infiltrates and the significant upregulation of MHC-II expressing cells, suggesting that activation of microglia may occur in an MHC-II-dependent manner and involve TG6-targeting humoral inflammatory responses in GA. Of note, the upregulation of MHC-II is not just a feature secondary to degeneration as it was not observed in the ataxia control group. Additionally, the two GA patients who were not on a GFD measured the highest levels of MHC-II immunoreactivity, which were associated with shorter disease duration but higher degree of pathological burden on haematoxylin and eosin stain. This observation supports the potential contribution of the GFD to dampening neuroinflammatory processes and might pose an explanation as to why sparse CD4 immunoreactivity was observed here: in the absence of dietary gluten as an autoimmunity trigger, the activation of CD4^+^ T cells is suppressed. Therefore, future research stratifying patients based on dietary habits and gluten intake throughout life could provide greater insight into the neuropathological outcomes of gluten exposure in GA, and studies investigating stage-dependent microglial activation are needed to better understand the correlation between microglial activation and the degree of disease. Interestingly, the presence of CD8^+^ cells observed in this study confirm the findings of Rouvroye *et al*.,^27^ pointing towards the potential existence of cytotoxic inflammatory processes in the cerebellum of GA patients. As mentioned previously, MHC-I-restricted CD8+ T cells contribute to cerebellar degeneration in PCD and their access into the CNS might be dependent on α4β1-integrin signalling.^51^ Recognition of gliadin peptides by Class I-restricted CD8^+^ T lymphocytes has been proposed previously in CD patients,^52^ and the involvement of MHC-I CD8+ T-cell responses in other autoimmune diseases appears to be disease stage specific.^53-55^ Therefore, it may be possible that similar cytotoxic responses participate in GA pathogenesis in a disease stage-dependent manner, explaining why we have observed higher loads of CD8^+^ cells in the two cases that displayed the most severe cerebellar pathology. However, immunoreactivity to CD8 was more extensive than CD3 immunoreactivity and little overlap was observed for CD3 and CD8 positivity. CD8 is also expressed on microglia/macrophages,^56,57^ and natural killer cells can present a CD3^−^CD8^+^ surface phenotype.^58,59^ Our study is limited by the PM material available for analysis, which prevented us from assessing any further correlations between MHC-II expression and the presence of lymphocytic inflammatory infiltrates and from determining whether the variation in MHC-II expression reported here directly correlates to the presence of a heightened immune response sustained by ongoing exposure to gluten. Further work is needed to investigate whether low CD4 immunoreactivity is indeed a feature of the GA brain and to confirm the nature of CD8^+^ cells observed here and to further characterize their subset.

Perivascular deposits of anti-TG6 antibodies have been reported in the cerebellum of GA patients,^6^ along with TG6 expression by mouse PCs.^60^ Additionally, TG6 shares TG2’s capacity to deamidate/transamidate peptides harbouring the most common gliadin T-cell epitopes known for CD^20^ and has been demonstrated to autocatalytically form isopeptide bond-linked immunogenic TG6–gliadin complexes.^18^ It is, therefore, conceivable that stable immune complexes harbouring both gliadin T-cell epitopes and TG isozyme are circulating and could mediate extra-intestinal immune activation. Such circulating antigen–antibody complexes have been reported for TG3 in dermatitis herpetiformis.^61^ The extravasation of lymphocytes into the perivascular space and neuropil reported in this study may indicate dysfunction of the blood–brain barrier as a result of cell-mediated inflammation, which in turn could facilitate the entry of gut-derived immune complexes. As a result, the MHC-II^+^ microglia observed in the cerebellum of GA patients could be involved in the presentation of immune complex-derived gluten T-cell epitopes. Tertiary lymphoid structures could develop and lead to the consequential formation of anti-TG6 and anti-gliadin antibodies intrathecally, which could interact with the resident TG6 enzyme. Alternatively, IgA-producing plasma cells have been shown to migrate out of their niche and into the CNS in an attempt to regulate neuroinflammation in a mouse model of experimental autoimmune encephalomyelitis.^62^ Furthermore, gut re-colonization of germ-free mice leads to an increase in meningeal plasma cells, whose B-cell receptor sequencing has confirmed their intestinal origin.^63^ However, mature antibody-secreting plasma cells normally downregulate CD20^64^ and TG2-specific plasma cells in the gut of CD patients have been shown to be CD20^−65^. Perivascular CD20^+^ cells may be immature plasmablast precursors that have been identified in the circulation in response to antigen challenge.^64^ Future work should aim to identify whether TG6–gliadin complexes are present in the circulation and investigate the presence of anti-TG6 and anti-gliadin IgGs/IgAs in CSF from patients with GA to better understand whether these antibodies are produced intrathecally or originate from outside the CNS. Further histological characterization is needed to confirm the presence of BBB leakage and to investigate whether the lymphocytes observed in the present study are fully differentiated plasma cells, plasmablast precursors or plasmacytoid lymphocytes.

The microgliosis observed in the superior cerebellar peduncles could be secondary to the degeneration of the cerebellum, as they represent the principal efferent pathway connecting the deep cerebellar nuclei to higher cortical structures.^66^ Additionally, microgliosis in the dorsal column was a prominent feature of the spinal cord, supporting the notion that amongst GA patients, up to 40% show symptoms of sensorimotor axonal peripheral neuropathy, with degeneration of the posterior column of the spinal cord.^2^ Interestingly, an unusual pattern of immunoreactivity to Iba-1 was present in one case in which Iba-1^+^ and MHC-II^+^ staining was present towards the surface of the spinal cord, potentially indicating a diffusion gradient driving inflammation from the CSF. CSF-restricted oligoclonal bands and pleocytosis have been reported in rare cases of rapidly progressive forms of GA.^67^ However, further research is needed to provide an in-depth characterization of CSF in patients with GA, including inflammatory markers and the presence of anti-TG6 and anti-gliadin antibodies to better understand the mechanisms of autoimmunity and cerebellar involvement in GA.

Iba-1 has long been considered a pan-microglial marker, although one that is not primarily associated with microglial activation^68^ but rather microglial motility.^69^ On the other hand, CD68 stains the lysosomal compartment of cells of the macrophage lineage. Its expression is associated with an increase in phagocytic activity,^70^ and it has been suggested that not all CD68^+^ microglia are Iba1^+^.^71^ This evidence could provide a potential explanation for the decrease in Iba-1-expressing cells observed in the parietal cortex of non-gluten-sensitive ataxia patients as a potential indicator of a shift in microglial phenotype towards a more phagocytic one and therefore CD68 dependent. However, no significant differences in CD68 expressing cells were measured in the parietal cortex of non-gluten-sensitive ataxia patients compared with the other groups, and therefore, further research is needed to conclude whether microglia lose their Iba-1 expression and what might be driving these changes and the exact nature of the respective cell population. In contrast, the significant upregulation in CD68 expression in the pons of the non-gluten-sensitive ataxia group accords with the current understanding of the neuropathology of genetic and degenerative forms of ataxia such as cerebellar variant of multiple system atrophy and spinocerebellar ataxia type 2. In cerebellar variant of multiple system atrophy and spinocerebellar ataxia type 2, the pontine basis and the corticospinal tracts are the areas mostly affected by degeneration, together with the cerebellum and middle cerebellar peduncles.^72-75^ This could explain the upregulation in CD68 expressing cells we measured in the pons in the non-GA ataxia control group as a potential response to axonal degeneration and synaptic loss.^76,77^

## Conclusion

In conclusion, we are the first to demonstrate that the cerebellum is the CNS region with the highest degree of pathological burden in GA, consistent with the primary clinical features. The pathology is characterized by atrophy with subtotal loss of PCs, infiltration of inflammatory lymphocytes and significant upregulation of microglial MHC-II expression in GA. Although the immunological mechanisms behind GA remain to be addressed by future research, our findings provide evidence for an immune-driven neuroinflammatory component involved in GA pathogenesis and highlight the importance of early diagnosis and treatment with GFD as well as the potential use of immunosuppressive therapies that could halt the neurodegenerative consequence of such inflammation.

## Supplementary Material

fcae078_Supplementary_Data

## Data Availability

Data from the study are available from the authors on reasonable request.
