# Supplementary material for: Cerebellar degeneration in gluten ataxia is linked to microglial activation
Source: Brain Commun. 2024 Mar 7;6(2):fcae078. doi: 10.1093/braincomms/fcae078 (PMC10953628; doi:10.1093/braincomms/fcae078)
Supplement: fcae078_Supplementary_Data [file fcae078_supplementary_data.docx]

**Supplementary Information**

Gluten ataxia is associated with cerebellar degeneration and microglial activation; A neuropathological study

**Supplementary Data**

Supplementary Fig.1 to 3

Supplementary Table 1 to 5


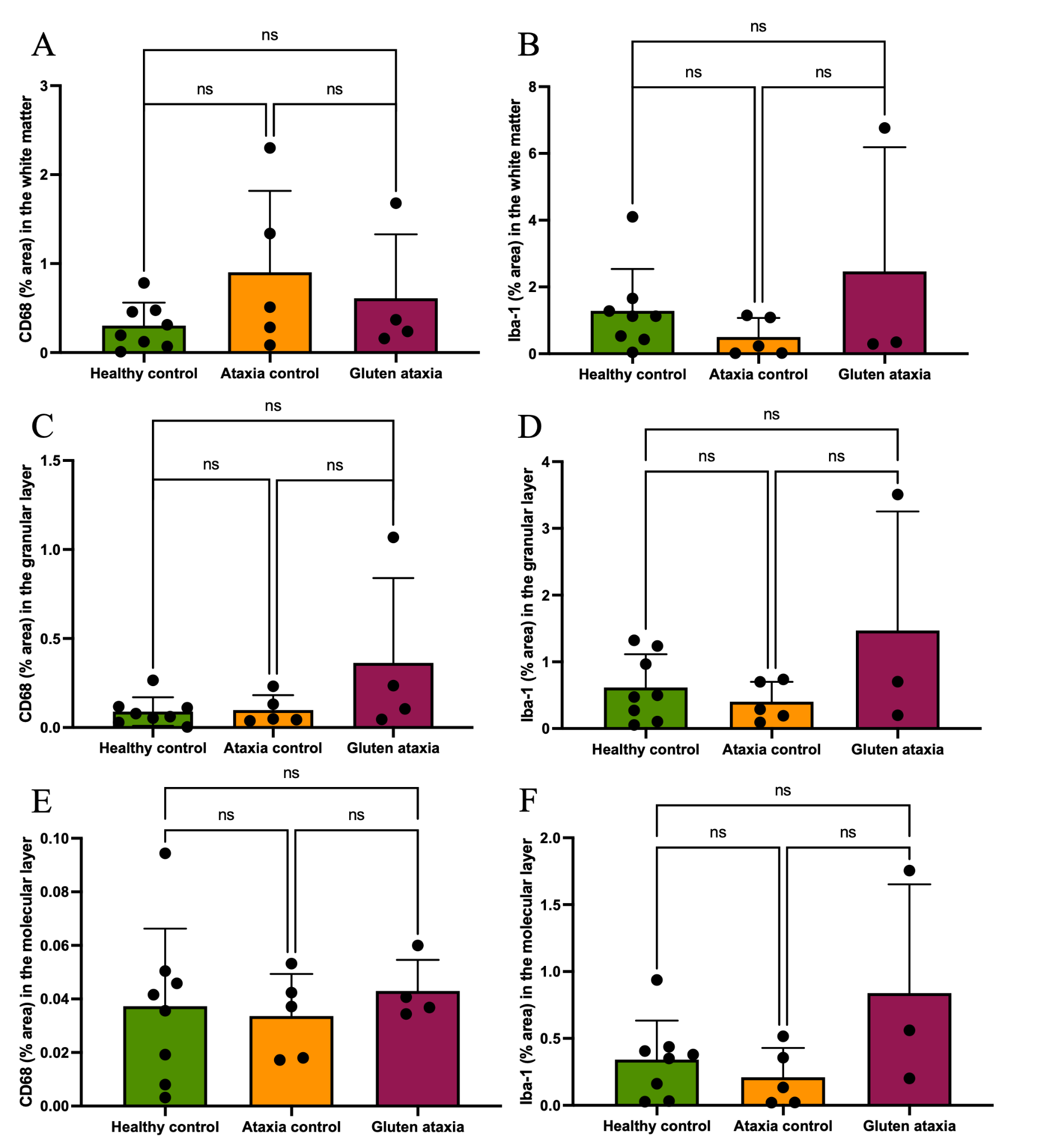


**Supplementary Figure 1 Immunoreactive profile of CD68 and Iba-1 in the cerebellar cortex and white matter across the cohort.** A non-significant increase in CD68 expression in the GL (*p*=0.8049) (C) and in Iba-1 expression in the WM (*p*=0.9844) (B), GL (*p*=0.8749) (D) and the ML (*p*=0.2056) (F) of GA cases relative to ataxia controls was observed. Significance determined by non-parametric Kruskal-Wallis test followed by Dunn’s multiple comparison post hoc test. All tests were performed 2-tailed and significant *p* values were <0.05.


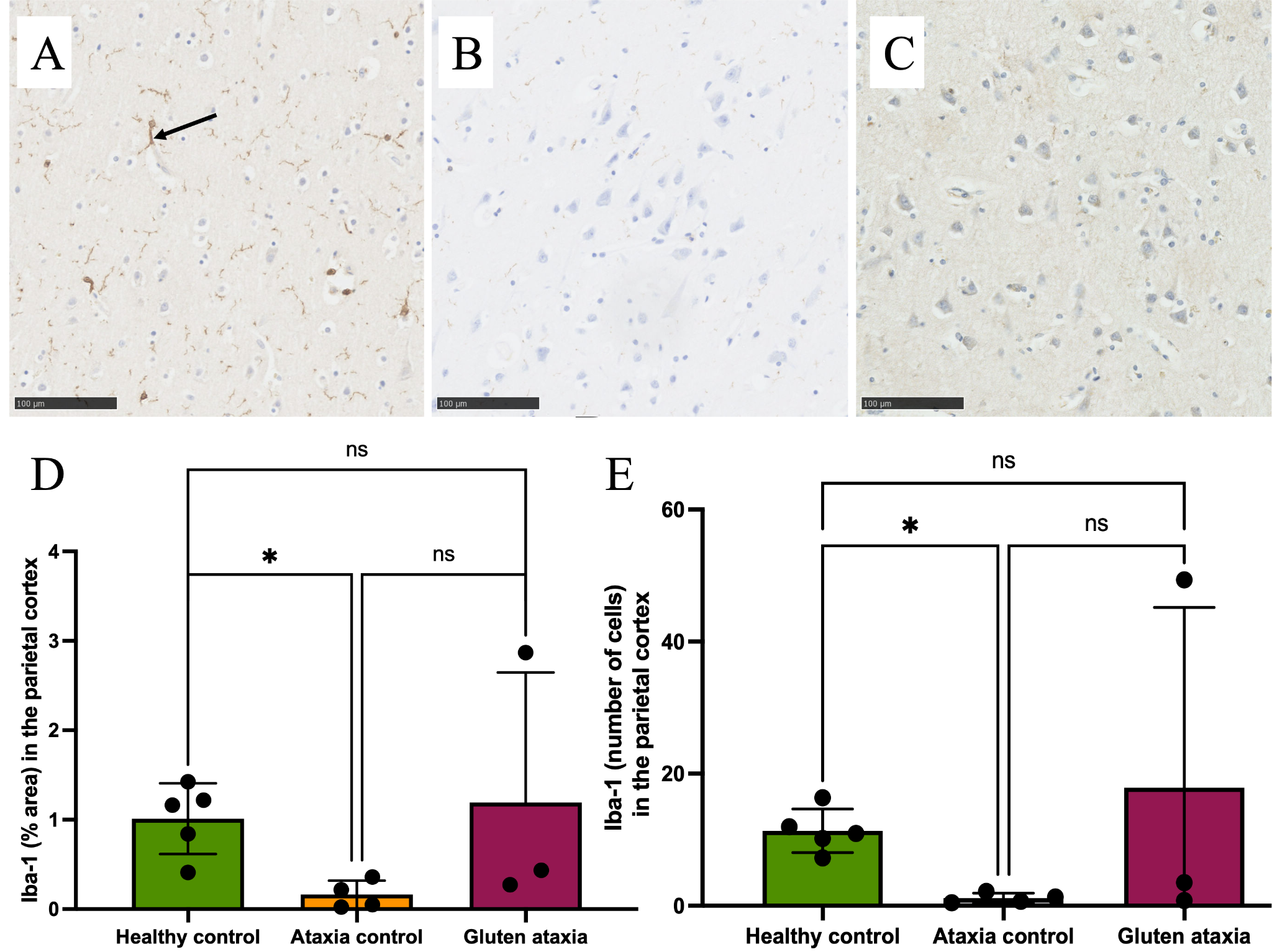


**Supplementary Figure 2 Immunoreactive profile of Iba1 in the parietal cortex across the cohort.** The HC group displayed the presence of ramified microglia positive to Iba-1 in the parietal cortex (arrow in A), which were rarely observed in the AC group (B) or GA (C). A significant decrease in Iba-1 immunoreactivity was measured in the parietal cortex of the ataxia control group compared to neurologically healthy controls (*p*=0.0371 for % area; *p*=0.0393 for number of cells) but not to GA cases (*p*=0.2226 for % area; *p*=0.4390 for number of cells). Significance determined by non-parametric Kruskal-Wallis test followed by Dunn’s multiple comparison post hoc test. All tests were performed 2-tailed and significant *p* values were <0.05.

*Scale bar represents 100µm (A-C).*


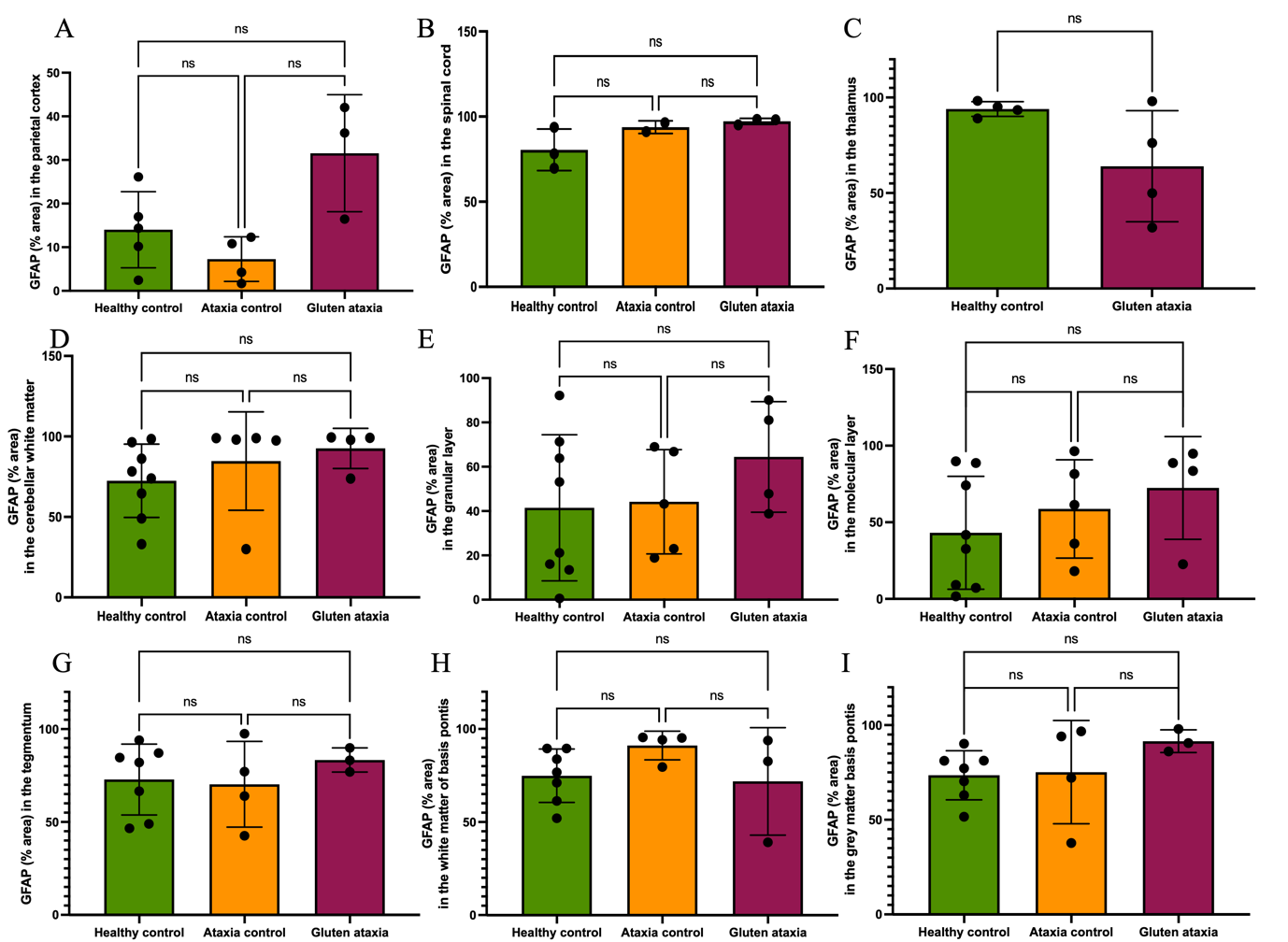


**Supplementary Figure 3 Immunoreactive profile of GFAP across the CNS.** A non-significant increase in GFAP expression was detected in the parietal cortex of GA patients relative to ataxia controls (*p*=0.0505), but not to healthy controls (*p*=0.4057) (A). No significant differences were detected in the spinal cord (B), thalamus (C), cerebellum (D-F) or the pons (G-I). Significance determined by non-parametric Kruskal-Wallis test followed by Dunn’s multiple comparison post hoc test. All tests were performed 2-tailed and significant *p* values were <0.05.

**
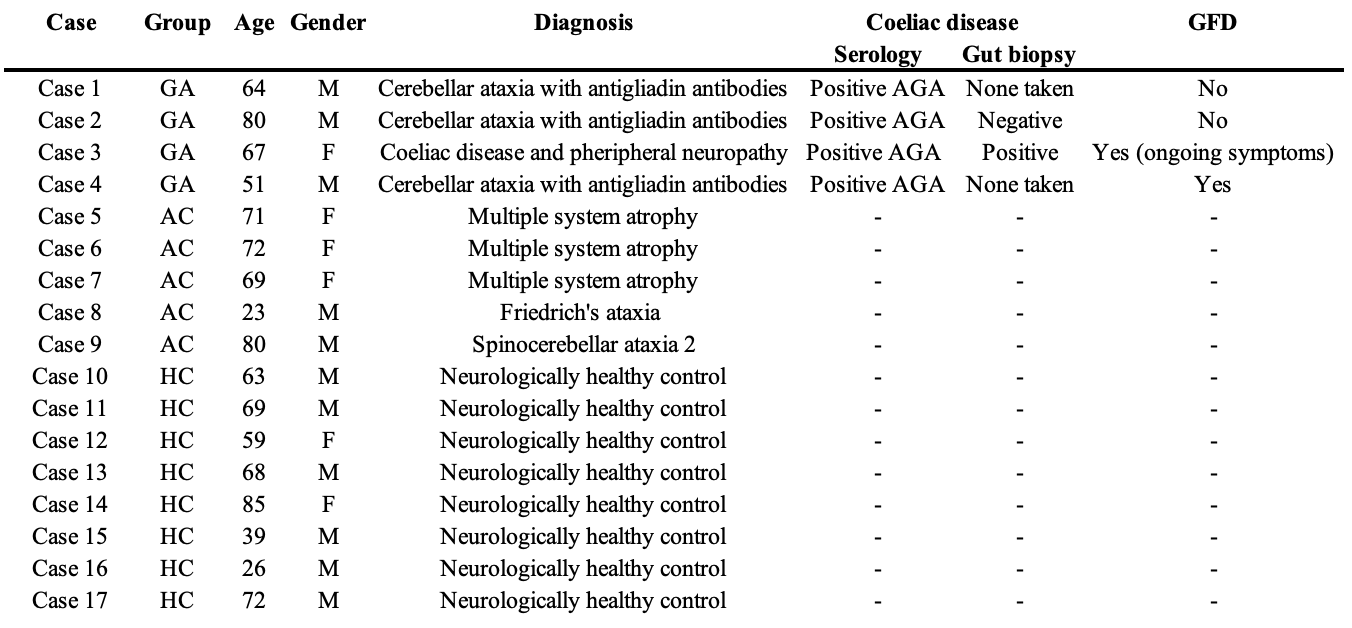
**

**Supplementary Table 1 Demographic table of study participants.**


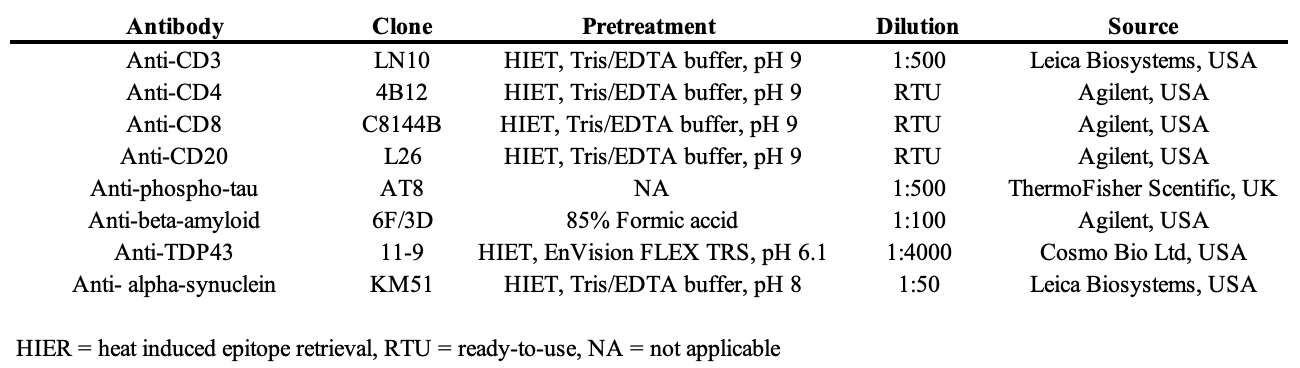
**Supplementary table 2 Optimal conditions for T-cell, B-cell and neurodegenerative markers**


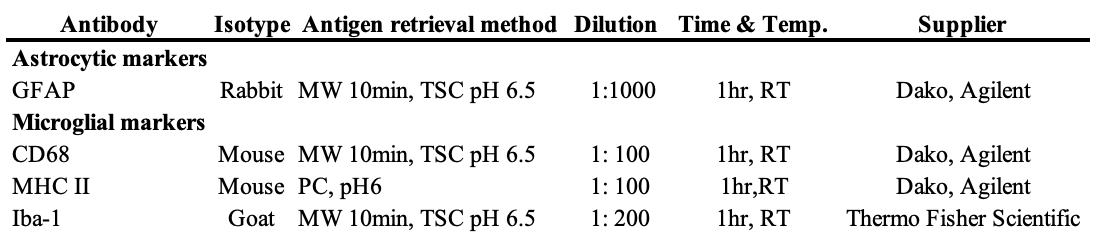


**Supplementary** **Table 3 Optimal conditions for antibody panel.** GFAP= Glial Fibrillary Acidic Protein; CD68= Cluster of differentiation 68; MHC-II= Major Histocompatibility Complex II; Iba-1= Ionised Calcium-binding Adaptor Protein 1; MW=microwave; PC=pressure cooker; TSC= trisodium citrate

**
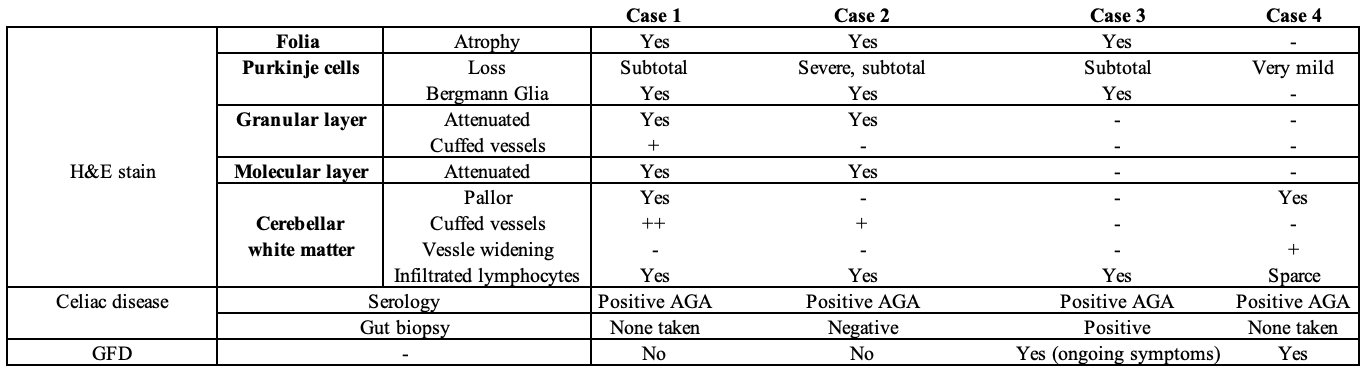
**

**Supplementary table 4 Histological, immunological and serological findings in GA cases.** Percentage of blood vessels showing pathological changes: + 10-25%; ++ 25-50%, +++ 50-75%. Celiac disease pathology as evidence by serological markers and gastroscopy. Information of GFD adherence at the time of death. GFD = gluten-free diet.


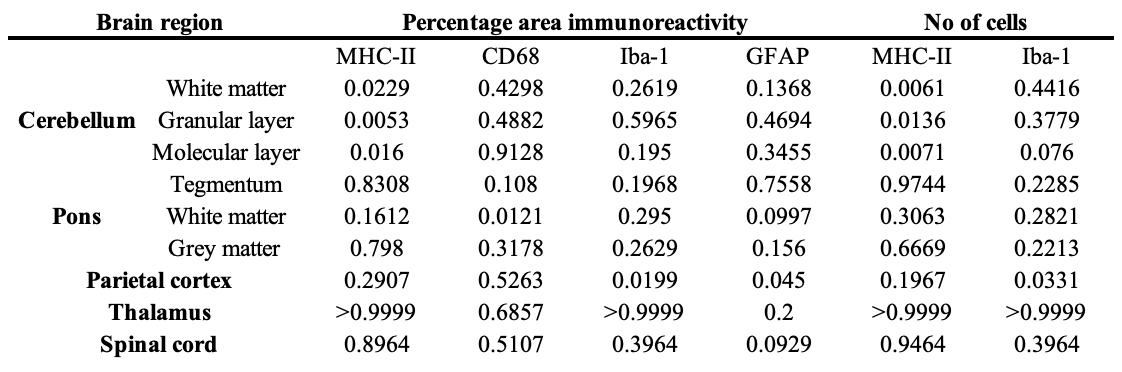
 **Supplementary Table 5 Inferential statistics of a panel of neuroinflammatory markers.** P-values after Kruskal-Wallis test across the three groups investigated for the percentage area immunoreactivity and number of immunoreactive cells for each marker of interest.
